# Cerebellum and Cognition Henrietta Leiner's contribution. Historical note

**DOI:** 10.1590/s1980-5764-2016dn1004017

**Published:** 2016

**Authors:** Hélio Afonso Ghizoni Teive, Walter O. Arruda

**Affiliations:** 1 MD, PhD. Movement Disorders Unit, Neurology Service, Internal Medicine Department, Hospital de Clínicas, Federal University of Paraná, Curitiba, PR, Brazil.; 2 MD, MSc. Movement Disorders Unit, Neurology Service, Internal Medicine Department, Hospital de Clínicas, Federal University of Paraná, Curitiba, PR, Brazil.

**Keywords:** cerebellum, cognition, language

## Abstract

The authors present the scientific contribution of Professor Henrietta C. Leiner, one of the pioneering scientists in the study of cognitive function of the cerebellum.

## Abstract (pt)

Os autores apresentam a contribuição científica da Professora Henrietta C. Leiner, um dos cientistas pioneiros no estudo da função cognitiva do cerebelo.

## INTRODUCTION

The cerebellum (from Latin, little brain) is situated in the posterior fossa of the skull, posteriorly to the brainstem and is composed of three parts: an unpaired, median portion called the vermis, and two lateral masses known as the cerebellar hemispheres.^1^ Phylogenetically, the cerebellum has been divided into three parts, the archicerebellum (vestibular cerebellum), the paleocerebellum (spinal cerebellum) and the neocerebellum (cortical cerebellum), that has connections with the motor brain cortex.^1,2^ The cerebellum has several connections via afferent and efferent fibers with the brain, brainstem and spinal cord.^1,2^ The so-called cortico-pontine-cerebellar-dentate-rubro-thalamic-cortico-pyramidal circuitry denotes the main cerebellar connections.^1,2^ Traditionally, the cerebellum is intrinsically associated with motor coordination, and its dysfunction promotes the clinical picture of ataxia. Cerebellar ataxia is defined by the presence of several signs, including dysmetria, dysdiadochokinesia, kinetic tremor, dysarthria (slurred speech), nystagmus, gait ataxia, hypotonia, pendular deep reflexes, and the presence of Holmes rebound phenomenon.^1,2^ The cerebellum's role in cognitive functions was studied by a number of researchers and culminated with the classical study of Schmahmann, published in1998, defining that cerebellum lesions, particularly involving the posterior lobe and vermis, can cause the "Cerebellar cognitive affective syndrome".^3^ This cognitive and affective syndrome is characterized by impairment of executive functions such as planning, set-shifting, verbal fluency, abstract reasoning and working memory; difficulties with spatial cognition including visual-spatial organization and memory; personality change with blunting affect or disinhibited and inappropriate behavior; and language deficits including agrammatism and dysprosodia.^3^ The consequences of cerebellar dysfunction on cognition and affect was defined by Schmahmann as the "dysmetria of thought".^4^ However, prior to Schmahmann's study, other researchers had made a major scientific contribution, calling attention to the link between the cerebellum and cognitive functions.^5^ In this historical review, we discuss the outstanding contribution of Professor Henrietta Leiner in this area.

## HENRIETTA C. LEINER – PRELIMINARY STUDIES

In 1940, Henrietta Leiner, a prominent scientist, was recruited by the U.S. government, at that time involved in World War II. She had expertise in both mathematics and physics, and was hired as a mathematical analyst at the National Bureau of Standards, to work on a secret electronic device related to the war.^6^ After that, she worked on a project to develop an electronic computer, and studied information-processing mechanisms, such as the human brain.^6^ In 1960, Henrietta Leiner and her husband, Alain L. Leiner, went to New York to work at the IBM research center.^6^ She was then accepted into the Columbia University, in the medical school, and admitted to an elementary course in neural anatomy, coordinated by Professor Charles R. Noback.^6^ During the dissection of the human brain, she saw a massive tract of nerve fibers descending from the cerebral cortex to the bottom of the brain, where the cerebellum was located. She became very puzzled by the famous human tract, and after that she started progressive research in this area.^6^ One important question posed by Leiner was "*Why would the cerebral cortex send so much high-level information down to the "low-level" cerebellum?*" ^6^ In 1970, the Leiner couple worked at IBM, in Palo Alto, California, USA, and Henrietta Leiner went to Stanford University. There she read a paper published by Professor Robert S. Dow, at that time an international authority on the cerebellum, who was working in Portland, Oregon. ^6,7^ After this, Henrietta Leiner and Dow started collaborative studies focusing on the link between the cerebellum and cognition.^8-12^ Henrietta C. Leiner and Alan L. Leiner subsequently published important papers studying the relationship between the cerebellum and cognition.^13^

## CEREBELLUM AND COGNITION – HENRIETTA LEINER'S CONTRIBUTION

The first contribution of Leiner's studies was the statement that the cerebellum is one of the most impressive parts of the human brain, and it had been underestimated for centuries.^6^ Interesting information came out of Leiner's first publication on this topic, in 1986, suggesting that the cerebellum contributes to mental skills.^8^ One of the mysterious questions was why the human cerebellum enlarged so dramatically in the last million years of human evolution. Additionally, the cerebellum sends projections of nerve tracts to enlarged association areas in the frontal lobe.^6^ On the other hand, the human cerebellum contains more neurons that the rest of the nervous system put together and can process information rapidly. One of the questions related to cerebellar involvement in cognitive and language functions could be explained by the dramatic human cerebellum enlargement.^6^ Leiner's hypothesis was that the cerebro-cerebellar circuitry of humans enables the cerebellum to improve the speed and skill of cognitive and language performance.^9-11^ Together Alan L. Leiner and Henrietta Leiner defined the cerebellum as "*The treasure at the bottom of the brain*".^6^ One important conclusion of Leiner's studies was that "*the cerebellum couples the motor function of articulating speech to the mental function that selects the language to be spoken, thus helping to produce fluent human speech and language*".^6^ In the paper entitled "Solving the mystery of the human cerebellum" Leiner stated that "*the combination in the cerebellum of motor and mental capabilities enables the cerebellum to confer on humans some adaptive advantages of great value, and this ability would explain why the human cerebellum has continued to enlarge dramatically*". ^6^

**Figure 1:**
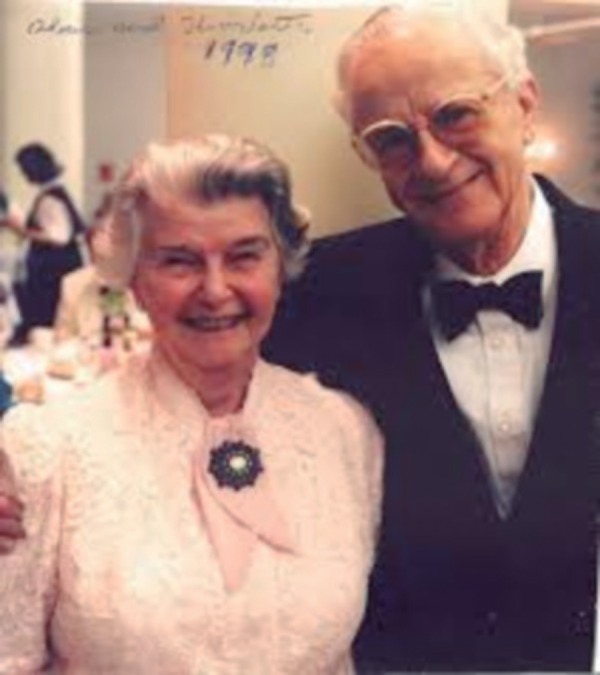
Professor Henrietta C. Leiner and her husband Alan L. Leiner (1998). (Extracted from Google Images: link springer.com, September, 18^th^, 2016).

## CONCLUSION

Nowadays, the cerebellar cognitive affective syndrome is well-known worldwide, demonstrating the nonmotor manifestations of cerebellar lesions in patients. It is very important to remember the seminal contributions of Professor Henrietta C. Leiner in this area. As a mathematical analyst who worked in computer systems, she studied neuroanatomy and similarities between brains, particularly the cerebellum, and machines, emphasizing the cerebro-cerebellar learning loops in humans, and ultimately the cerebellum's contribution to cognitive functions. ^6,8-12^

## References

[1] Arruda WO, Meneses MS (2011). Cerebelo. Neuroanatomia aplicada.

[2] Manto MU, Manto MU (2010). Embriology and anatomy. Cerebellar disorders. A practical approach to diagnosis and
management.

[3] Schmahmann JD, Sherman JC (1998). The cerebellar cognitive affective syndrome. Brain.

[4] Schmahmann JD (1998). Dysmetria of thought: clinical consequences of cerebellar
dysfunction on cognition and affect. Trends Cogn Sci.

[5] Schmahmann JD (2010). The role of the cerebellum in cognition and emotion: Personal
reflections since 1982 on the dysmetria of thought hypothesis, and its
historical evolution from theory to therapy. Neuropsychol Rev.

[6] Leiner HC (2010). Solving the mystery of the human cerebellum. Neuropsychol Rev.

[7] Dow RS (1995). Cerebellar cognition. Neurology.

[8] Leiner HC, Leiner AL, Dow RS (1986). Does the cerebellum contribute to mental skills?. Behav Neurosci.

[9] Leiner HC, Leiner AL, Dow RS (1987). Cerebro-cerebellar learning loops in apes and
humans. Ital J Neurol Sci.

[10] Leiner HC, Leiner AL, Dow RS (1991). The human cerebro-cerebellar system: its computing, cognitive,
and language skills. Behav Brain Res.

[11] Leiner HC, Leiner AL, Dow RS (1993). Cognitive and language functions of the
cerebellum. Trends Neurosci.

[12] Leiner HC, Leiner AL, Dow RS (1989). Reappraising the cerebellum: what does the hindbrain contribute
to the forebrain?. Behav Neurosci.

[13] Leiner HC, Leiner AL (1997). How fibers subserve computing capabilities: similatities between
brains and machines. Int Rev Neurobiol.

